# Gut Microbiome in Older Men: Findings From the Osteoporotic Fractures in Men Study

**DOI:** 10.1093/geroni/igaa057.3073

**Published:** 2020-12-16

**Authors:** Michelle Shardell, Lisa Langsetmo, Ryan Demmer

## Abstract

There is great interest in identifying determinants and health consequences of the human gut microbiota, the dynamic population of microorganisms living in the human digestive tract. However, the role of gut microbiota in the health of older adults has received considerably less attention than that among younger or middle-aged adults. Findings among younger age groups are not necessarily generalizable to older adults due to differences in lifestyle, health conditions, and medication usage. Therefore, understanding the role of gut microbial communities in aging-related phenotypes is an emerging gerontology research priority. To fill this significant knowledge gap, the Osteoporotic Fractures in Men (MrOS) Study Microbiome Ancillary Study was conducted in 2014-2016, coinciding with the parent study’s 4th clinic visit. A total of 912 men with mean aged 84.2 (standard deviation=4.0) years provided fecal samples, and 16S ribosomal RNA target gene sequencing was used to characterize the gut microbiota composition. In this symposium, we present findings on the first research projects completed with these data. Dr. Lisa Langsetmo will characterize the association between objectively measured physical activity and the composition of gut microbiota. Dr. James Shikany will present dietary patterns, another lifestyle determinant of gut microbiota. Dr. Deborah Kado will focus on a specific micronutrient, vitamin D, and its metabolites as another factor that may influence the gut microbiota in older men. Lastly, Dr. Michelle Shardell will overview the analytical challenges of microbiome research and illustrate an approach to quantify the potential role of gut microbiota composition and weight in older men.

